# Repetitive transcranial magnetic stimulation for stroke rehabilitation: insights into the molecular and cellular mechanisms of neuroinflammation

**DOI:** 10.3389/fimmu.2023.1197422

**Published:** 2023-05-22

**Authors:** Rongjun Sheng, Changchun Chen, Huan Chen, Peipei Yu

**Affiliations:** ^1^ Department of Radiology, The First People’s Hospital of Linping District, Hangzhou, China; ^2^ Department of Radiology, The People’s Hospital of Qiandongnan Miao and Dong Autonomous Prefecture, Guizhou, China; ^3^ Department of Radiology, The People’s Hospital of Longyou, Quzhou, China; ^4^ Department of Radiology, Sanmen People’s Hospital, Taizhou, China

**Keywords:** transcranial magnetic stimulation, stroke, rehabilitation, neuroinflammation, microglia, neurotransmitter, neuroimaging technique

## Abstract

Stroke is a leading cause of mortality and disability worldwide, with most survivors reporting dysfunctions of motor, sensation, deglutition, cognition, emotion, and speech, *etc.* Repetitive transcranial magnetic stimulation (rTMS), one of noninvasive brain stimulation (NIBS) techniques, is able to modulate neural excitability of brain regions and has been utilized in neurological and psychiatric diseases. Moreover, a large number of studies have shown that the rTMS presents positive effects on function recovery of stroke patients. In this review, we would like to summarized the clinical benefits of rTMS for stroke rehabilitation, including improvements of motor impairment, dysphagia, depression, cognitive function, and central post-stroke pain. In addition, this review will also discuss the molecular and cellular mechanisms underlying rTMS-mediated stroke rehabilitation, especially immune regulatory mechanisms, such as regulation of immune cells and inflammatory cytokines. Moreover, the neuroimaging technique as an important tool in rTMS-mediated stroke rehabilitation has been discussed, to better understanding the mechanisms underlying the effects of rTMS. Finally, the current challenges and future prospects of rTMS-mediated stroke rehabilitation are also elucidated with the intention to accelerate its widespread clinical application.

## 1 Introduction

Stroke is one of the leading causes of mortality and disability worldwide, with most survivors reporting a decrease in quality of life, placing heavy financial burden on patients’ families and society (1, 2). The stroke can lead to an imbalance in blood supply and thereby induce severe brain damages, result in various dysfunctions, such as motor impairment, dysphagia, cognition impairment, post-stroke pain, depression, *etc (* 3). Current best stroke treatment is to minimize initial brain damage and prevent subsequent complications, and then improve function through rehabilitation training (4). Moreover, most stroke miss the best therapeutic window for emergency treatment, such as thrombolysis or thrombectomy, therefore, the rehabilitation is quite important for stroke patients to improve motor, swallowing, and cognitive functions (5, 6). Currently, lots of rehabilitation programs have been applied by rehabilitation specialist to recovery the impaired functions and improve the quality of life. Commonly used rehabilitation programs in clinical practice include: physical therapy, occupational therapy, speech therapy, hyperbaric oxygenation, acupuncture, *etc (* 7), which can improve post-stroke dysfunction by stimulating central nervous system (CNS) input through sensorimotor system or by promoting CNS remodeling through intensive training of motor patterns. At present, these traditional rehabilitation programs remain the main methods for post-stroke recovery, which can promote the recovery of various dysfunctions after stroke to a certain extent. However, there are also some shortcomings for these rehabilitation programs, such as slow onset, time-consuming, and poor patient compliance (8). Due to the certain shortcomings of traditional rehabilitation programs, various novel techniques have been utilized for stroke rehabilitation, such as repetitive transcranial magnetic stimulation (rTMS), transcranial direct current stimulation (tDCS), virtual reality, augmented reality, brain-computer interface, and rehabilitation robot, which have shown great stroke rehabilitation effects (9–12).

rTMS, as a noninvasive brain stimulation (NIBS), is a neurostimulation technique that uses a pulsed magnetic field to regulate the membrane potential of neurons, thereby selectively modulating the neural excitability of brain regions (13). rTMS has been applied in neurological and psychiatric diseases, including cognitive impairment, depression, mental disease, and Alzheimer’s disease (AD) (14–17). It is painless and easy to operate, making it a valuable tool for providing more precise brain area regulation and prognostic assessment in stroke rehabilitation. The interhemispheric inhibition (IHI) model is the primary theoretical basis for the application of rTMS in stroke rehabilitation (18). This model suggests that the two brain hemispheres are connected by the corpus callosum, which not only transmits information between the hemispheres but also regulates their interactions. Under healthy condition, the excitatory or inhibitory activity between the two hemispheres is balanced through the corpus callosum. However, stroke disrupts the balance of mutual inhibition between the hemispheres, resulting in weakened inhibition of the affected hemisphere on the unaffected hemisphere, and increased inhibition of the unaffected hemisphere on the affected hemisphere (4). Another model is called compensation, which suggests that the neural conduction in the damaged area is disrupted after stroke (19). The neurons and astrocytes in the unaffected hemisphere can form new circuits to compensate for the damaged area, allowing for the recovery of motor function in stroke patients. However, the compensation model cannot be applied when a patient experiences a bilateral stroke (20). A lager number of clinical trials have been conducted to evaluate the potential of rTMS to promote rehabilitation of limb, swallowing, and cognitive functions post-stroke, which showed satisfactory recovery outcomes (21–24).

In this review, the clinical benefits of rTMS for stroke rehabilitation are summarized, including improvements of motor impairment, dysphagia, depression, cognitive function, and central post-stroke pain. Besides, this review will also focus on the molecular and cellular mechanisms underlying rTMS-mediated stroke rehabilitation, especially those related to immune regulation.

## 2 Clinical benefits of rTMS for stroke rehabilitation

rTMS have been shown to promote effectively rehabilitation of neurological sequelae post-stroke, including motor impairment, dysphagia, cognitive impairment, mental diseases, and neuropathic pain, which will be summarized and discussed in this section (**Table 1**).

**Table 1:** Main clinical outcomes after rTMS with various characteristics.

Study	Stroke stage	rTMS site	rTMS frequency (Hz)	Intensity (%)	Combined treatment	Clinical outcomes	Outcome measures	References
Aşkın et al. (2017)	Chronic (n=40)	Contra-M1	1	90 RMT	Physical therapy	Improvement of upper limb motor function	FMA, BBT, FIM, FAS	(25)
Lüdemann-Podubecká et al. (2016)	Subacute (n=10)	Contra-PMd	1	110 MT	/	Improvement of motor function of affected hand	JTT, BBT, MEP, CSP, ISP	(26)
Tosun et al. (2017)	Acute/subacute (n=25)	Contra-M1	1	90 RMT	Physical therapy/NMES	Improvement of upper limb motor function	BRS, FMA, fMRI, UE-MI, BI, MAS	(27)
Hosomi et al. (2016)	Subacute (n=41)	Ipsi-M1	5	90 RMT	/	Improvement of motor function of paralytic hand	BS, NIHSS, FMA, FIM	(28)
Sasaki et al. (2017)	Acute (n=21)	bilateral leg motor areas	10	90 RMT	/	Improvement of lower limb motor function	BRS, ABMS II	(29)
Choi et al. (2016)	Chronic (n=30)	motor cortical area of the 9^th^ thoracic erector spinae muscles	10	90 RMT	/	Improvement of balance function	BBS, CDP	(30)
Cheng et al. (2014)	Chronic (n=4)	Ipsi-tongue motor cortex	5	90 RMT	/	Improvement of swallowing functions and swallowing related quality of life	SAPP, VFSS, TPA	(24)
Khedr et al. (2009)	Acute (n=26)	Ipsi-oesophageal cortical area	3	100 RMT	/	Improvement in dysphagia	BI, MEP, Grip strength	(31)
Sasaki et al. (2017)	Chronic (n=13)	Region spanning from the dACC to mPFC	10	80 RMT	/	Improvement of apathy	QIDS, AS	(32)
Sharma et al. (2020)	Subacute (n=96)	Contra-M1	1	110 RMT	Physical therapy	Improvement of motor function	HAMD, mBI, mRS, FMA, NIHSS	(33)
Kim et al. (2010)	Chronic (n=18)	Left DLPFC	1, 10	80 MT	/	Improvement of mood	BDI, CPT, mBI	(23)
Yin et al. (2020)	Chronic (n=34)	Left DLPFC	10	80 RMT	/	Improvement of cognitive function and quality of life	ALFF, FC, mBI	(34)

### 2.1 Motor impairment

Most stroke patients experience upper limb motor impairment, with only 5%-20% of them being able to fully recover their upper limb function (35). Upper limb impairment can significantly affect stoke patients’ abilities, leading to a negative impact on their quality of life (36). For example, The loss of upper limb function can severely affect tasks such as eating, dressing, and personal hygiene care, and result in a loss of independence (37). Stroke disrupts the balance between the brain hemispheres, which is an important cause of upper limb motor impairment after stroke (38, 39). Repetitive transcranial magnetic stimulation (rTMS) can modulate cortical excitability and thus recovery the balance post-stroke (40, 41). According to IHI model, there are two main options to using rTMS to promote functional recovery post-stroke: one is to use low-frequency (≤1Hz) rTMS (LF-rTMS) to stimulate the unaffected hemisphere and reduce its excitability, thus decreasing its inhibitory effect on the affected hemisphere; the other is to use high-frequency (≥3Hz) rTMS (HF-rTMS) to stimulate the affected hemisphere and increase its excitability, thereby restoring balance between the hemispheres (42–44). Numerous studies have revealed that rTMS over primary motor cortex (M1) can improve upper limb motor function post-stroke (25, 26, 28, 43, 45, 46). A meta-analysis of Hsu et al. included 392 stroke patients from 18 studies, which suggest that rTMS could promote upper limb motor recovery in stroke patients, especially those with subcortical stroke (47). And they found that applying LF-rTMS on the unaffected hemisphere might be safer and more effective than HF-rTMS on the affected hemisphere in improving upper limb motor function after stroke. The evidence-based guidelines on the therapeutic use of rTMS, updated by International Federation of Clinical Neurophysiology in 2019, recommended that LF-rTMS over M1 of unaffected hemisphere at the subacute stage of stroke as level A evidence (definite efficacy) could recover hand motor effectively (48). HF-rTMS over the M1 of affected hemisphere at the subacute stage as well as LF-rTMS over M1 of unaffected hemisphere at the chronic stage of stroke were recommended as level B (probable efficacy) and C (possible efficacy) evidences. Consistent with the guideline, a meta-analysis of Mu group including 904 stroke patients from 34 studies indicated that the effectiveness of rTMS on stroke patient present timing-dependent manner: the acute phase > the subacute phase > the chronic phase (49). In addition, rTMS can be combined with other therapies to improve upper limb function after stroke, including occupational therapy (22), virtual reality training (50), action observation (51), and upper-limb training (52), *etc.*

Motor impairment increases the risk of falling due to gait impairments, resulting in limitations in activities of daily living and a lower quality of life (46, 53). Clinicians prioritize the improvement of lower limb motor function and walking ability when treating stroke patients (54, 55). Therefore, the study of rTMS on lower limb motor function also has significant clinical implications. Tung et al. performed a meta-analysis based on 169 stroke patients and found that rTMS could remarkably improve walking speed, lower limb activity, and Fugl-Meyer Assessment lower limb scores (56). Another meta-analysis from Li group indicated that rTMS, especially HF-rTMS over unaffected hemisphere, could significantly improve walking speed of stroke patients (57). In addition, the meta-analysis conducted by Vaz et al. revealed that either HF-rTMS or LF-rTMS combined with other rehabilitation therapies could significantly improve gait speed in both acute/subacute and chronic stages of stroke (58). In addition to improving walking speed, Choi et al. found that HF-rTMS [10 Hz, 90% resting motor threshold (RMT)] over the trunk motor cortex could remarkably improve the balance function of chronic stroke patients without any side effects (30).

Post-stroke lower limb spasticity impairs gait and balance to reduce speed of walking, thereby increasing the need of wheelchair and caregiver (59). A meta-analysis of Liu et al., including 554 stroke patients from 9 studies, aimed to evaluate the efficacy of rTMS in improving post-stroke lower limb spasticity (60). They revealed that rTMS could decrease Modified Ashworth Scale (MAS) score and elevate Modified Barthel Index (MBI) score, compared with the control group. Further subgroup analysis concluded that LF-rTMS showed a positive effect on lower limb spasticity after stroke, while the HF-rTMS showed no significant effect on the lower limb motor function. Due to the limited studies included in this meta-analysis, the recovery effect of HF-rTMS on lower limb spasticity remains to be studied. Besides, the mechanism by which rTMS improves lower limb spasticity is still unclear and needs to be further explored in future studies.

### 2.2 Dysphagia

Dysphagia is a common complication in stroke patients, with a prevalence of ~53%, which can result in aspiration pneumonia, malnutrition, electrolyte imbalances, and even death (61). Dysphagia is associated with prolonged hospital stay, poor life quality, elevated mortality, make it imperative to prioritize early intervention for improving swallowing function (62, 63). Current treatments include postural interventions, surgery, botulinum toxin injections and exercise, but these are less effective (64–66). Recently, noninvasive neurostimulation therapies have been found to improve the dysphagia in stroke patients (67). Among the noninvasive neurostimulation therapies, rTMS might be the most effective treatment for dysphagia after stroke, compared to tDCS, pharyngeal electrical stimulation (PES), and surface neuromuscular electrical stimulation (sNMES) (67). For instance, Khedr et al. evaluated the recovery effect of rTMS on the swallowing performance in 26 patients with dysphagia at the acute stage of stroke (31). In rTMS-treated group, the esophageal cortex of the affected hemisphere was received HF-rTMS daily (3 Hz and 120% of RMT) for 5-10 days. The results showed that the rTMS significantly improved the dysphagia of stroke patients for several months. In another study, Verin et al. applied LF-rTMS (1 Hz) on the mylohyoid cortical area of the unaffected hemisphere in patients with dysphagia at the chronic stage of stroke, resulting in a greater improvement in swallowing function and a remarkable decrease in the aspiration score for liquids (68). Besides, HF-rTMS (5 Hz) applied over the tongue region of the motor cortex of the unaffected hemisphere has been shown to improve swallowing performance of stroke patients with chronic dysphagia (24). Although the rTMS has been shown to improve swallowing function in a large number of studies, there is no unified treatment standard for its stimulation site, intensity and treatment duration (69–71). Therefore, it is necessary to conduct large-scale multi-center clinical studies on rTMS for dysphagia, and further develop standardized treatment guideline in the future.

### 2.3 Depression

Post-stroke depression (PSD) is the most common neuropsychological complication of stroke with an incidence rate of ~33% (72). PSD has been shown to reduce quality of life, affect rehabilitation outcome, and increase mortality rate (32, 73, 74). Current therapies for PSD include pharmacotherapy, psychotherapy and physical therapy, however, some patients do not benefit from these first-line treatments (75–77). Fortunately, several studies indicated that rTMS was expected to improve the neuropsychological disorder (78, 79). And the U.S. Food and Drug Administration (FDA) approved rTMS over left dorsolateral prefrontal cortex for treatment of the major depressive disorder (MDD) in 2008 (80). Thus, a large number of clinical trials have explored the therapeutic efficacy of rTMS on the PSD (81, 82). However, the results from these clinical trials were inconsistent. Thus, a meta-analysis of Shen et al. included 1764 PSD patients from 22 randomized controlled trials (RCTs) studies to explore the therapeutic efficacy of rTMS for PSD (83). The results showed that rTMS could remarkably improve the PSD measured by Hamilton Depression Rating Scale (HAMD). Further safety evaluation showed no statistical difference in withdrawals owing to adverse events. However, most of these results are from single studies with varying degrees of limitations. Therefore, definitive conclusions about the treatment of PSD with rTMS need to be further confirmed by multicenter RCTs. The traditional rTMS protocol requires treatment 5 days per week for more than 4 weeks. Frey et al. proposed an accelerated rTMS strategy to reduce the number of days needed to complete treatment, which can bring convenience to the patients and increase compliancy. They applied HF-rTMS (20 Hz) at 110% RMT over the left dorsolateral prefrontal cortex of the PSD patients 5 sessions per day for 4 days. The results revealed that HAMD of PSD patients remarkably reduced after the accelerated rTMS, which maintained for 3 months. Besides, no significant adverse events associated with rTMS treatment were observed in the study. This study suggested that the accelerated rTMS protocol could be a more convenient adjuvant treatment option for PSD patients.

### 2.4 Cognitive function

Post-stroke cognitive impairment (PSCI) occurred in nearly 75% of stroke patients (84). Only half of them are able to recovery the cognitive function, whereas the others might develop vascular dementia (85). Since stroke patients with PSCI may experience impaired judgment and memory problems, PSCI can hinder physical recovery (86). Furthermore, prolonged cognitive impairment can significantly affect activities of daily living (ADL), quality of life, and reintegration into the community (87–89). Thus, effective intervention to improve PSCI is a very important part of stroke rehabilitation. The therapies of PSCI included pharmacological therapy (*e.g.*, acetylcholinesterase inhibitor, memantine, traditional Chinese medicines, *etc.*), cognitive training, risk factor prevention and intervention (90, 91). In recent years, rTMS has been applied to treat cognitive impairment induced by several CNS diseases, including Alzheimer’s disease, depression, Parkinson’s disease, and bipolar disorder (14, 15, 92, 93). Notably, several studies have found that rTMS also showed positive therapeutic effects on PSCI (23, 34, 94–96). For example, Yin et al. applied 20 sessions of rTMS (10 Hz, 80% RMT) over the left dorsal lateral prefrontal cortex of PSCI patients, which revealed that rTMS could improve cognitive function and ADLs of PSCI patients (34). Moreover, the functional MRI (fMRI) of rTMS-treated patients indicated that the rTMS might activate left medial prefrontal cortex and augmented the functional connectivity to right medial prefrontal cortex and ventral anterior cingulate cortex, resulting in the improvements of cognitive function. These studies suggested that the rTMS can be an important and effective treatment to rescue the cognitive function in PSCI patients, and that functional connectivity (FC) and neural activity in cognition-related brain regions could be crucial indicators to clarify the effect of rTMS on PSCI. In addition, rTMS has exhibited a superior modulating effect in cognitive function compared to other non-invasive stimulation techniques (96). Intermittent theta-burst stimulation (iTBS) as a type of TMS has also been applied to improve the PSCI. Tsai et al. evaluated the therapeutic effect of rTMS (5 Hz, 80% RMT) and iTBS on the PSCI patients, suggesting that both rTMS and iTBS could effectively improve cognitive impairment, including global cognitive function, memory function, and attention (96). Compared with the iTBS group, the rTMS group showed better improvement in attention.

### 2.5 Central post-stroke pain

Central post-stroke pain (CPSP) is a neuropathic pain syndrome that can occur after a cerebrovascular accident, with an incidence rate of 1~12% (97, 98). The main treatment for CPSP is pharmacology (99). However, the pharmacology has limited pain control and unpleasant side effects. Recently, several studies found that HF-rTMS could relieve CPSP to some extent (100–102). Leung et al. found that rTMS showed great analgesic effect on CPSP (16.7% of visual analog scale score reduction), and rTMS exhibited better improvement in CPSP than peripheral neuropathic pain (101), suggesting that rTMS might be a promising therapeutic tool for CPSP.

FMA, Fugl–Meyer Assessment; BBT, Box and Blocks Test; FIM, Functional Independence Measurement; FAS, Functional Ambulation Scale; rTMS, repetitive transcranial magnetic stimulation; PMd, dorsal premotor cortex; RMT, resting motor threshold; MT, motor threshold; JTT, Jebsen–Taylor Hand Function Test; MEP, motor evoked potential; CSP, cortical silent period; ISP, ipsilateral silent period; NMES, neuromuscular electrical stimulation; fMRI, Functional magnetic resonance imaging; BRS, brunnstrom recovery stage; UE-MI, Upper extremity motricity index; BI, barthel index; MAS, Modified ashworth scale; BS, Brunnstrom stages; NIHSS, National Institutes of Health Stroke Scale; ABMS II, Ability for Basic Movement Scale Revised; ipsi, ipsilateral; contra, contralateral; BBS, Berg Balance Scale; CDP, Computerized dynamic posturogphy; SAPP, Swallowing Activity and Participation Profile; TPA, tongue pressure assessment; VFSS, Videofluoroscopic swallowing study; QIDS, Quick Inventory of Depressive Symptomatology; AS, Apathy Scale; dACC, dorsal anterior cingulate cortex; mPFC, medial PFC; mBI, modified Barthel Index; HAMD, Hamilton Depression Scale; mRS, modified Rankin Scale; DLPFC, dorsolateral prefrontal cortex; BDI, Beck Depression Inventory; CPT, Continuous Performance Test; ALFF, amplitude of low-frequency fluctuation; FC, functional connectivity.

## 3 The molecular and cellular mechanisms of neuroinflammation underlying rTMS-mediated stroke rehabilitation

**Figure 1:**
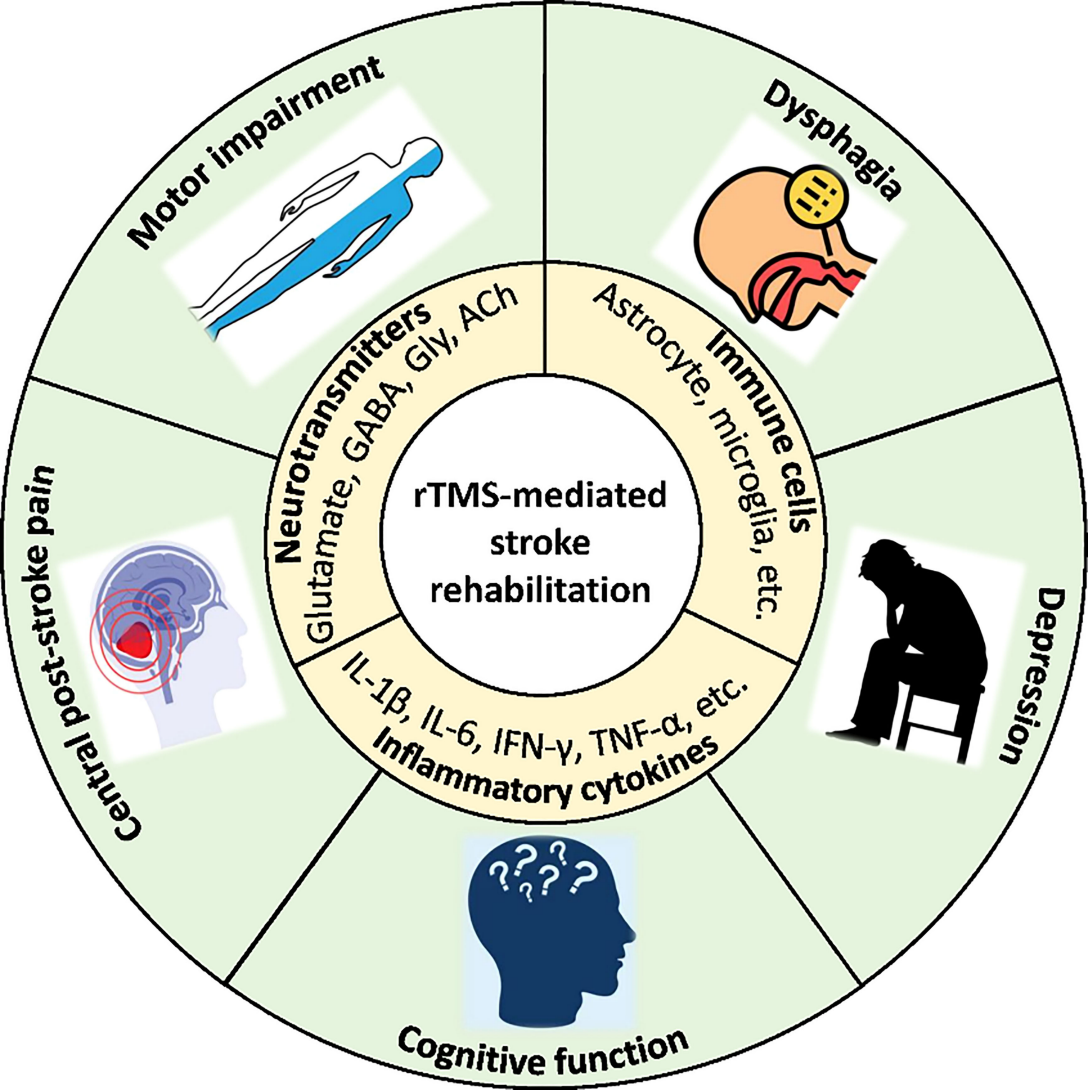
Scheme of neuroinflammation mechanisms of rTMS-mediated stroke rehabilitation.

Despite the confirmed value, the regulatory mechanism *via* which TMS shows the beneficial effects on stroke rehabilitation remain unclear. Recent studies have shown that the basic mechanisms might be involved in regulation of neurotransmitters release, immune cells, and cytokines (**Figure 1**) **(** 102–105).

### 3.1 Neurotransmitters

Neurotransmitters as chemical messengers are released from a neuron to excite or inhibit other neurons (106). Neurotransmitters, including glutamate, gamma-aminobutyric acid (GABA), glycine (Gly), and acetylcholine (ACh), play an important role in chemical synapses of the CNS. Thus, the abnormal metabolism or release of the neurotransmitters can result in synaptic dysfunction, impaired neurogenesis, impairment of cognitive function, depression, memory deficits and CPSP, *etc (* 107–109). Numerous studies have shown that stroke could induce abnormal release of neurotransmitters (107, 110). Therefore, modulation the neurotransmitters might be a rational approach to promote the stroke rehabilitation.

A number of studies have shown that rTMS could alter the release and expression of neurotransmitters in the CNS, which might be the underlying mechanisms of rTMS-based stroke rehabilitation (102, 105, 111–114). As an excitatory neurotransmitter, glutamate-mediated excitotoxicity is a crucial mechanism resulting in post-stroke injury (115). GABA and glycine are major inhibitory neurotransmitters in the brain (116). Ikeda et al. revealed that application of rTMS (20 Hz) were able to regulate the mRNA expression levels of several neurotransmitter-related genes, including GABAergic, glutamatergic, and glycinergic neurotransmission systems in mouse cerebellum and brain stem, suggesting that rTMS can modulate the activity of neurons and synaptic plasticity *via* regulating the levels of neurotransmitters (111, 114). Zangen et al. revealed that TMS over the frontal or caudal cortex of the healthy rat brain elevated the extracellular dopamine and glutamate levels in the nucleus accumbens (117). However, LF-rTMS (1 Hz) on the primary motor cortex of healthy human could not influence the excitatory (glutamate) neurotransmitter but decrease the inhibitory (GABA) neurotransmitter in both ipsilateral and contralateral motor cortices (112). Moreover, Chen et al. reported that rTMS (10 Hz) at the affected M1 and rTMS (1 Hz) at the unaffected M1 could reduce the GABA content in M1 of ischemic stroke patients, which was associated with the improvement of motor function (105). Several studies have shown that decreased GABAergic neurotransmission in the CNS might be a main cause of chronic neuropathic pain, such as CPSP (118). Decrease of inhibitory GABAergic tone was found at the level of dorsal spinal cord, somatosensory cortex, and thalamus sensory nuclei, which led to neuronal hyperactivity in the sensorimotor cortex (119). Besides, the neuronal hyperactivity related to deafferentation pain was caused by abnormal recruitment of N-methyl-D-aspartate (NMDA) receptors (120). Lefaucheur et al. found that HF-rTMS (10 Hz) on CPSP patients could significantly increase intracortical inhibition (ICI) which could reflect GABAergic neurotransmission function (113). Overall, the CPSP was associated with the imbalance between GABAergic and glutamatergic transmission in the CNS, which could be restored through rTMS-mediated GABAergic neurotransmission regulation.

Cholinergic neurons can synthesize and release the Ach when they are excited. Ach is an excitatory neurotransmitter and plays a key role in learning and memory processes (121). Li group treated the vascular dementia (VD) rats with rTMS (0.5 Hz, 1.33T) for 30 days and found that the rTMS treatment could remarkably enhance the acetylcholinesterase (AChE) and choline acetyltransferase (ChAT) activities, and increase the density of cholinergic neurons (122). The learning and memory deficits of VD rats was also significantly improved, the underlying mechanism of which might be associated with recovery of cholinergic nervous system activity in CA1 region of the hippocampus. Furthermore, brain-derived neurotrophic factor (BDNF), a neurotransmitter modulator, can promote the migration and proliferation of neural stem cells, and further enhance stroke recovery. Luo et al. revealed that HF-rTMS could activate BDNF/tropomyosin-related kinase B (TrkB) signaling pathway and antiapoptotic pathways to increase number of BrdU+ NESTIN+ cells, decrease Bcl-2 expression and elevate Bax expression, leading to the improvement of the cognitive impairment of rats with ischemic stroke (123). In addition, Cambiaghi et al. found that HF-rTMS was able to enhance dendritic complexity and spine number of neurons through BDNF and calcium-dependent signaling pathways (124).

### 3.2 Immune cells

Previous researches on the mechanism of rTMS were mainly concentrated on the effects on neurons. The HF-rTMS enhances neuronal excitatory at the affected hemisphere, while LF-rTMS decreases neuronal excitatory at the unaffected hemisphere (125). Nevertheless, little is known about the influence of rTMS on the other neural cells, such as astrocyte and microglia.

Astrocytes are the most abundant glial cells and play an important role in the maintenance of the blood-brain barrier (BBB) and CNS homeostasis (126). The astrocytes are activated after stroke and further polarized into two distinct phenotypes: neurotoxic (pro-inflammatory) type A1 and neuroprotective (anti- inflammatory) type A2. A1 astrocytes can secrete inflammatory cytokines and neurotoxic mediators to induce neuroinflammation and further aggravate brain damage, while A2 astrocytes mainly secrete anti-inflammatory cytokines and nerve growth factor to promote neuroregeneration and exert neuroprotective functions. In addition, astrocytes have neurotransmitter receptors and ion channels, which play a crucial role in synaptic neurotransmission (127). Therefore, rTMS might be able to alter cell membrane potential and further regulate the function of astrocytes (128, 129).

rTMS with different frequencies has been shown to regulate the expression of GFAP (a cytoskeletal marker of astrocytic reactivity) and the density of GFAP-positive astrocytes in various *in vivo* brain injury or disease model, such as cortical stab injury, Parkinson’s disease, *etc (* 130–132). Moreover, Clarke et al. found that rTMS (1 or 10 Hz) over the culture mouse cortical astrocytes could alter the expression of genes and proteins related to calcium signaling and inflammation, such as intercellular adhesion molecule 1 (Icam1), stromal interaction molecule 1 (Stim1), and ORAI calcium release-activated calcium modulator 3 (Orai3), *etc (* 133).

Astrocyte modulation has also been indicated as one mechanism for rTMS-mediated stroke rehabilitation. Hong et al. evaluated the effects of rTMS on astrocyte polarization in *in vitro* and *in vivo* cerebral ischemic/reperfusion injury model (134). They revealed that the rTMS (10 Hz) could decrease the concentration of pro-inflammatory cytokine TNF-α, increase anti-inflammatory cytokine IL-10, and regulate astrocytic polarization (from A1 to A2 astrocytes) after ischemic/reperfusion injury. Besides, the astrocyte culture medium collected from rTMS-treated astrocytes could remarkably reduce ischemic/reperfusion injury-induced neuronal apoptosis. In addition, the rTMS could suppress the excessive astrocyte-vessel interactions and promote the vasculature-associated A1 to A2 astrocyte switch in the peri-infarct cortex after stroke, which was beneficial to vessel and BBB protection post-stroke (103). Further mechanistic studies displayed that rTMS significantly upregulated the expression level of platelet-derived grow factor receptor beta (PDGFRβ) which mediated the interactions of A2 astrocytes and their adjacent vessels, and that the angiogenesis-associated factors (TGFβ and VEGF) in A2 astrocytes were remarkably increased. Overall, these results suggested that rTMS could reduce neuroinflammation, inhibit neuronal apoptosis, and protect the vasculature by regulating astrocyte polarization, thus promoting stroke rehabilitation.

Microglia are the resident immune cells of the CNS, which are distributed in the gray matter and white matter, accounting for ~10% of the number of cells in the brain (135). Microglia maintain a resting state with certain migration and swallowing ability when not activated. They are able to monitor the CNS microenvironment and remove the necrotic neurons timely, thereby maintaining the CNS homeostasis (136). Once activated, microglia can polarize into two distinct phenotypes: the pro-inflammatory M1 and the anti-inflammatory M2 (137). In addition, microglia are early participants in post-stroke neuroinflammation and play an important role in the post-stroke recovery stage (138, 139). Shifting the balance of microglial polarization towards the anti-inflammatory M2 phenotype has been shown to exhibit neuroprotective effects in cerebral stroke (140–142). However, the influence of rTMS on the microglia has been largely unexplored. The number or state of microglia in motor cortex of healthy rats was not changed following application of chronic LF-rTMS (1 Hz) (143). Nevertheless, application of HF-rTMS on the Mongolian gerbils with cerebral ischemia has been shown to significantly increase the number of activated microglia and the expression of Iba1 in the hippocampus (144). Zong et al. applied HF-rTMS (50 Hz) over the affected hemisphere of rat photothrombotic (PT) stroke model, which significantly improved behavioral functions and infarct volume post-stroke (104). Microglial over-activation has been shown to affect neuroinflammation, leading to neuronal death. The immunofluorescence results displayed that Iba1 immunoactivity was obviously increased in the peri-infarct region of PT stroke rat, which could be remarkably rescued by rTMS treatment. Besides, they found that rTMS could effectively induce a M1 to M2 switch in microglial phenotypes, as evidenced by downregulation and upregulation of proteins related to M1 activation (CD74, CD32, and CD86) and the M2 phenotype (CD206, IL-10, and IL-4), respectively. Furthermore, the reactive microglia can release signals to promote astrocytic activation and polarization (145). The A1 to A2 switch in astrocytic phenotypes was also found in rTMS-treated PT stroke rat (103). Luo et al. revealed that rTMS could induce the anti-inflammatory M2 phenotype of microglia and facilitate the generation of anti-inflammatory cytokines (146). Moreover, they found that neural stem cells cultured with medium from rTMS-treated microglia presented decreased apoptosis and increased neuronal differentiation. In addition, Chen et al. revealed that HF-rTMS could regulate the Janus kinase 2 (JAK2)-signal transducer and transcription 3 (STAT3) pathways, and further inhibit microglial activation as well as promote the switch of microglia toward the neuroprotective M2 phenotype, resulting in alleviation of ischemic white matter damage and improvement of cognitive impairment (147). Taken together, rTMS may regulate the microglial polarization and further modulate the inflammatory microenvironment, thereby promoting neurogenesis and improving stroke rehabilitation.

### 3.3 Inflammatory cytokines

Previous studies have shown that the expression levels of inflammatory cytokine, including interleukin (IL)-1β, IL-2, IL-6, IL-10, IL-17a, interferon (IFN)-γ, transforming growth factor beta (TGF-β), and tumor necrosis factor alpha (TNF-α), in brain tissues and peripheral blood were significantly changed after stroke (148, 149). Among these cytokines, IL-1β, IL-2, IL-6, TNF-α, and IFN-γ are pro-inflammatory, while the IL-10 and TGF-β belong to anti-inflammatory cytokines. IL-10 were reported to be secreted by several cell types after stroke, including M2 microglia, macrophages, regulatory T cells, and B lymphocytes. TGF-β, a factor secreted by A2 astrocytes which regulates cell growth and differentiation, can inhibit the activation of inflammatory/immune cells and the release of various inflammatory mediators, thereby promoting tissue repair after injury (150). Inflammatory cells, including microglia, astrocytes, and infiltrating peripheral immune cells (neutrophils, monocytes and macrophages, *etc.*), in the ischemic lesions after acute ischemic stroke can release a large number of pro-inflammatory cytokines, including TNF-α, IL-1β, and IL-6, *etc*, which can cause neuronal damage and upregulate the levels of selectin and ICAM-1 to increase the permeability of cerebral vascular endothelium (151, 152). Moreover, these pro-inflammatory cytokines can also recruit peripheral neutrophils, macrophages, and lymphocytes, which further amplifies the neuroinflammation, eventually causing a vicious cycle of pro-inflammatory. Additionally, the occurrence of PSD is closely related to the imbalance of serum levels of inflammatory cytokines, such as IL-2, IL-10, IL-17a and IFN-γ (153). Among various mechanisms of rTMS-mediated stroke rehabilitation, regulation of inflammatory cytokine may be one of the important mechanisms. Cha et al. revealed that HF-rTMS on the affected dorsolateral prefrontal cortex (DLPFC) of stroke patients could significantly reduce the mRNA level of pro-inflammatory cytokines (IL-1β, IL-6, TGF-β, TNF-α) in blood samples, indicating the anti-inflammatory effect of rTMS (154). Besides, the reduction of IL-6 was strongly correlated with increase of auditory verbal learning test (AVLT, r=-0.928) and complex figure copy test (CFT, r=-0.886). Another study on the ischemic stroke also found that the rTMS were able to decrease the serum levels of IL-6, IL-8, and TNF-α (155). Furthermore, the rTMS treatment could suppress the M1 microglia polarization *via* let-7b-5p/HMGA2/NF-κB pathway, and further decrease the TNF-α level but elevate the IL-10 concentration, promoting the anti-inflammatory effect (155). iTBS treatment could remarkably reduce the high concentrations of IL-1β, IFN-γ, TNF-α, and IL-17A as well as increase the IL-10 level in brain tissues of cerebral ischemic mice (156). In addition, the pro-inflammatory cytokines could affect the synaptic plasticity, which might further influence cognitive function and behavior (157–159). For example, the TNF-α, produced by microglia and peripheral immune cells, could affect long-term potentiation (LTP) of excitatory neurotransmission at higher concentration (160, 161). Therefore, the regulation of cytokine concentrations in serum and brain tissues is an important mechanism of rTMS-mediated rehabilitation, which could also be used as indicators to evaluate the effect of stroke rehabilitation.

## 4 The roles of imaging techniques in rTMS-mediated stroke rehabilitation

Imaging techniques play an important role in understanding the mechanisms underlying the effects of rTMS and in guiding the optimization of rTMS protocols. First, these imaging techniques could be applied to identify the optimal target. Specifically, imaging techniques such as fMRI and TMS mapping can help identify the regions of the brain that are affected by the stroke and the regions that need to be targeted by rTMS for optimal recovery (162). Secondly, the imaging techniques can be used to assess the treatment effects of rTMS on brain function and connectivity of stroke patients. For example, fMRI can be utilized to measure changes in brain activity, while diffusion tensor imaging (DTI) can be used to detect changes in white matter integrity (163, 164).Li et al. utilized resting-state fMRI (rs-fMRI) to explore the effect and mechanism of rTMS on PSCI patients (95). They revealed that the rTMS (5 Hz, 100% RMT) could significantly improve cognitive functions measured by Minimum Mental State Examination (MMSE) and Montreal cognitive assessment (MoCA). They also found that FC and neural activity in several cognition-related brain regions were significantly regulated by rTMS therapy. These imaging results indicated that the rTMS showed effective impact on the PSCI patients to improve their cognitive function. Besides, the imaging techniques can be applied to personalize rTMS treatment for individual patients. For instance, fMRI can be used to identify regions of the brain that are still functionally active after stroke, and then rTMS can be targeted to these regions to enhance recovery (165). In addition, the imaging techniques can be also utilized to predict the outcomes of rTMS-mediated stroke rehabilitation. The diffusion MRI can be used to predict motor function recovery after stroke, which could also guide rTMS treatment (166). Overall, imaging techniques are an important tool in rTMS-mediated stroke rehabilitation, providing valuable information on the underlying mechanisms of stroke recovery and helping to optimize rTMS protocols for individual patients.

## 5 Prospects and challenges

rTMS is a novel technique that can regulate neurotransmitters, activation/polarization of immune cells (astrocytes and microglia), and inflammatory cytokines in the brain, thereby affecting brain function and improving post-stroke dysfunctions. Moreover, rTMS combined with other traditional rehabilitation programs can present a synergistic effect and further enhance the rehabilitation effect of stroke patients.

According to previous safety evaluation, although the incidence rate is low, rTMS could induce several side effects, including epilepsy, syncope, short-term hearing loss, headache pain, dizziness, toothache, and paresthesia, *etc (* 167–172). Thus, the rTMS should be applied within the range of treatment parameters according to the guideline. There are many stimulation parameters of rTMS, including coil type, coil position, stimulation frequency, stimulation time, stimulation interval, and total stimulation volume, *etc*, which might be the main reason for the heterogeneity of clinical treatment effects and basic research results. Due to the obvious differences in different rTMS stimulations and clinical outcome evaluation indicators, it has brought great difficulties to the study of optimal stimulation parameters. In some clinical studies, patients in control group did not receive reliable sham stimulation, therefore, it is difficult to determine the relative efficacy of placebo in rTMS. Generally, the sample size of current clinical studies of rTMS-mediated stroke rehabilitation is still insufficient. Therefore, randomized, double-blind, large sample size, and placebo-controlled clinical studies should be performed in the future to further clarify the clinical value of TMS. In addition, LF-rTMS and HF-rTMS are more clinically applied at present. TBS has fewer clinical applications, but it requires lower stimulation intensity, fewer pulses, and produces longer-lasting cortical excitability compared to the formers. Thus, TBS might be a promising approach for stroke rehabilitation, which needs to further explored in the future study.

Recent study has shown that LF-rTMS on the frontal cortex could reduce the local inhibition and disrupt feedforward and feedback connections, whereas, the LF-rTMS on the occipital cortex could enhance the local inhibition and increase forward signaling. A more thorough understanding of the mechanism underlying the rTMS-mediated CNS regulation allow us to better apply rTMS to clinical treatment. However, the current mechanism research is still in the preliminary stage, thus, future studies need to further explore the regulatory mechanism of rTMS on the CNS, especially the regulation of immune inflammation.

## Author contributions

RS wrote the manuscript. PY drew the pictures. CC and HC revised the manuscript. RS and PY collected the literature. All authors contributed to the article and approved the submitted version.
